# Making an Inlay Matrix

**Published:** 1902-02-15

**Authors:** H. J. Bosart

**Affiliations:** Springfield, Ohio


### MAKING AN INLAY MATRIX.

BY H. J. BOSART, SPRINGFIELD, OHIO.

Read at Ohio State Meeting December 4, 1901.

The difficulty encountered in obtainiing a good inlay lies I think, in the difficulty in securing a perfect fitting matrix, one which will fit the cavity and retain its exact shape until it is invested and the baking is finished.

First prepare cavity for making matrix by trimming he edges to the extent necessary, leaving them perfectly square, sharp and smooth, avoiding undercuts, filling in overhanging edges or even leaving some of the more solid decay. After the matrix is completed you can then remove decay and undercut the cavity to retain the finished inlay. Number thirty gold foil makes the best matrix, as it is softer than platinum and being more easily molded to the irregularities of the cavity.

Select a piece of foil of sufficient size, and with a pellet of cotton work the gold into and well over the bottom of cavity, then hold and work out to the edges until the foil is perfectly fitted to the cavity, then bring the foil out over the tooth's surface, and hold with the fingers, or stick it to the tooth with Canada Balsam dissolved in chloroform. Place this also in the cavity of the matrix to facilitate removal and handling, either by the points of a broach with a slight bend forming a hook, or with a bit of floss silk with a knot at the end. Now fill the matrix with Hill's temporary stopping, using as much care as when putting in a gutta percha filling, the more care used in placing this filling, the more perfectly the matrix will be conformed to the shape of cavity. This latter hardening makes an unyielding support to the matrix, preventing the possibility of a change in shape during the process of removal from the cavity and the investing of the matrix.

By the aid of the previously inserted broach or floss silk, filling and matrix are easily removed and invested. Make the investment of plaster with water to the desired consistency and place in the baking tray sufficient to imbed the matrix, slightly tapping the tray to settle the matrix in the investment, allowing it to cover the free edges but not quite reach cavity line.

To insure expulsion of the air bubbles carefully coat the reverse side of matrix with the mixed investment plaster before imbedding.

When investment is hard, warm sufficiently to soften the temporary stopping which can then nearly all be removed by the broach or floss silk. What remains wash from matrix with chloroform. This will not soften or affect the investment. Then fill in the matrix with the selected enamel and bake in the usual manner, with the full assurance that the finished inlay will be a perfect fit.

